# Real-Time Flow Behavior of Hot Mix Asphalt (HMA) Compaction Based on Rheological Constitutive Theory

**DOI:** 10.3390/ma12101711

**Published:** 2019-05-27

**Authors:** Guoping Qian, Kaikai Hu, Xiangbing Gong, Ningyuan Li, Huanan Yu

**Affiliations:** 1 School of Traffic and Transportation Engineering, Changsha University of Science & Technology, Changsha 410114, China; guopingqian@csust.edu.cn (G.Q.); kaikaihu@stu.csust.edu.cn (K.H.); huanan.yu@csust.edu.cn (H.Y.); 2 Key Laboratory of Special Environment Road Engineering of Hunan Province, Changsha University of Science & Technology, Changsha 410114, China; 3 Department of Civil and Environmental Engineering, University of Waterloo, Waterloo, N2L 3G1, Canada; ningyuanli@hotmail.com

**Keywords:** hot mix asphalt, Nishihara model, viscoelastic-plastic, compaction process

## Abstract

Compaction is the most critical stage during pavement construction, but the real-time rheological behavior in the compaction process of hot mix asphalt has not received enough attention. Rheological properties directly reflect the of mixture performance, the intrinsic directly reflects the influencing factors of compaction, and the pavement compactness and service life. Therefore, it is important to interpret the rheological properties of the asphalt mixture during the compaction process. In this paper, the improved Nishihara model was used to study the viscoelastic-plastic properties of the hot mix asphalt in the compaction process. Firstly, the improved Nishihara model was briefly introduced. Subsequently, the stress and strain correlation curves are obtained by the MTS (Material Testing System) compaction test, and the strain-time curve is fitted to determine the model parameter values. Finally, the parameters are substituted into the constitutive equation to obtain the strain-time curve and compared it with the test curve. The results show that the improved Nishihara model effectively depicts the real time behavior of the asphalt mixture in the compaction progress. The viscos and plastic parameters present certain differences, which reflects that the gradation and temperature have certain influence on the compaction characteristics of the mixture.

## 1. Introduction

The compaction of asphalt pavement affects the performance of pavement structure and service life. Rutting, friable, pothole, and other failures usually occur when the pavement layers are not well compacted [1,2,3]. One of the reasons for the poor compaction is that there are no complete theories for the real time rheological properties of asphalt mixture under high temperature and non-compaction conditions. Therefore, it is necessary to establish an actual mechanical model to evaluate the deformation of the asphalt mixture during the construction compaction process.

Asphalt mixture is a kind of thermo-viscoelastic-plastic material; the flow behavior of hot mix asphalt is concluded to be directly related to the rheological properties. Its total strain includes elastic strain, viscoelastic strain, plastic strain, and viscoplastic strain [4,5,6]. Its mechanical properties are related to stress state, temperature, and loading time. There has been a lot of research that is aimed at using the constitutive model to predict the permanent deformation of asphalt mixture [7,8,9]. Various time-dependent constitutive models have been proposed in the past few decades [10,11]. Giunta et al. [12] established a constitutive model that consisted of the Maxwell model and hardened viscoplastic element to simulate the time-dependent non-linear behavior of asphalt mixtures. The model can reflect the experimental characteristics of the material and match thermodynamic requirements. Masad et al. [13,14] used the Perzyna viscoplastic model to describe the mechanical behavior of asphalt mixtures in constant strain rate compression tests. However, this model does not lack the ability to represent the recovery behavior during repeated loading-unloading cycles, because the viscoelastic model is not coupled to the viscoplastic model. Sun et al. [15] used the internal variable theory and the thermodynamic orthogonal principle to establish a continuous two-stage viscoelastic-viscoplastic constitutive model for simulate the aging characteristics of asphalt mixtures.

Other researchers have proposed some models to study the mechanical response of asphalt mixture under high temperature conditions. Huang et al. [16] proposed a temperature-dependent HiSS plastic model, which can reflect the nonlinear plasticity, temperature, and load rate of asphalt mixture. Darabi et al. [17] proposed a phenomenological rate-dependent hardening-relaxation model that was coupled with nonlinear Schapery viscoelastic and Perzyna viscoplastic models to accurately predict the permanent deformation of asphalt mixture under cyclic compression loads at high temperatures. However, they did not pay attention to radial strain, so the model could not accurately describe the three-dimensional mechanical properties of the asphalt mixtures. Pasetto et al. [18] established a viscoelastic-plastic constitutive model by viscoelastic elements and a slider that is connected by elastic springs in parallel with three Maxwell components to analyze the mechanical response and creep deformation ability of asphalt concrete at high temperatures. However, there is a lack of in-depth study regarding the creep mechanism of asphalt mixture and its relationship with viscoelastic response. Chen et al. [19] proposed a comprehensive constitutive model that is capable of capturing the main mechanical properties of asphalt materials based on the thermodynamic finite strain frame model, which can predict the complex time- and temperature-dependent response of asphalt materials during elongation and compression, and it successfully predicts the permanent deformation of the road.

The constitutive theory to predict the permanent deformation of asphalt mixture has been extensively studied and successfully used in practice. However, the rheological properties of the hot mix asphalt during compaction and the change law of viscoelastic-plastic parameters are seldom studied. Chen [20] established the viscoelastic-plastic constitutive relation of hot mix asphalt by Material Testing System (MTS) compaction test and SGC (Superpave Gyratory Compactor) gyratory compaction test, while using the basic principles and methods of viscoelastic plastic force. Liu [21] used the Bodnet–Partom model to simulate the rheological properties of asphalt mixture during compaction and simulated it with finite element software.

The above-mentioned models are complicated to solve the parameters; on the contrary, the Nishihara model is intuitive and theoretically simple, and it can clearly and comprehensively reflect the viscoelastic-plastic in the roller compaction. However, it does not consider the effects of nonlinearity and temperature stress. Therefore, based on the Nishihara model, this paper establishes a nonlinear Nishihara model to simulate the rheological behavior of the asphalt mixture during compaction. Through a MTS compaction test that was designed to simulate the construction compaction process, the rheological parameters were obtained, and the feasibility and rationality of the model were verified. The relation between compaction properties and model parameters was finally discussed based on the real-time parameter analysis.

## 2. Test Materials and Methods

**Figure 1:**
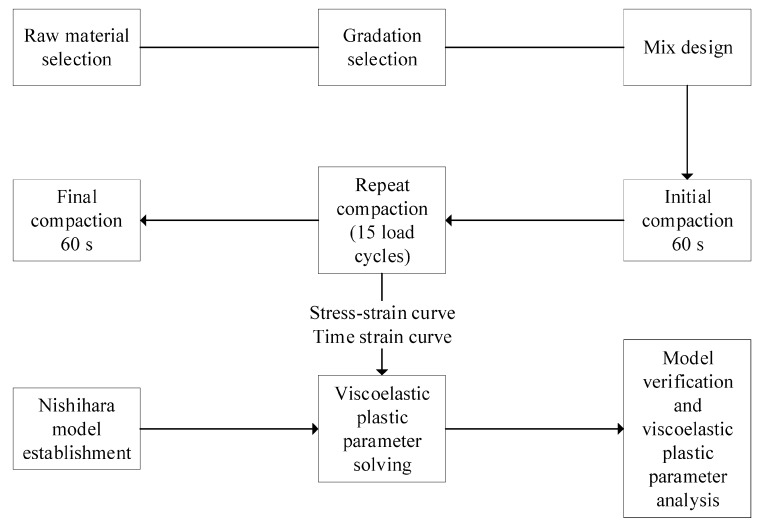
Flow chart of the materials and method used in this study.

This chapter describes the asphalt performance, mixture gradations, and MTS compaction test methods. The MTS compaction test is used to obtain the model parameters and verify the model. Figure 1 shows the flow chart of this article.

### 2.1. Materials and Mix Design

The asphalt is a CNOOC Taizhou 70# matrix asphalt, and Table 1 shows its physical properties. The aggregates used in this paper are basalt, and Table 2 and Table 3 shows the physical properties. Aggregates with the size larger than 2.36 mm are coarse aggregates, and the aggregates with the size smaller than 2.36 mm are fine aggregates. The mineral filler is ground limestone, and Table 4 shows the physical properties. All of the tests are requirement Chinese standards [22,23,24].

**Figure 2:**
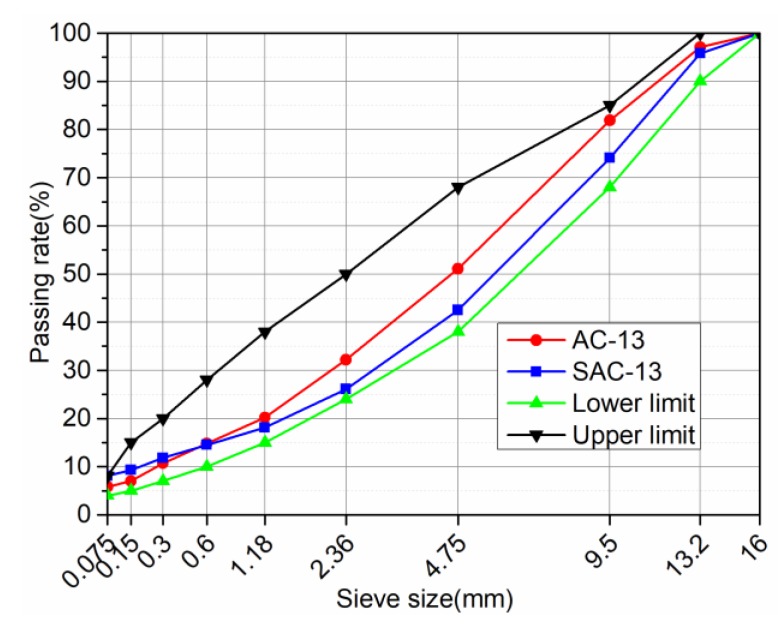
Gradation curves of AC-13 and SAC-13.

Two different types of asphalt mixtures were used in the test: Fine grain asphalt concrete (AC-13) and multi-gravel asphalt concrete (SAC-13). Figure 2 shows the gradation. Multi-gravel asphalt concrete (SAC-13) contains more coarse aggregates, which can provide deeper surface structure, smaller voids and less water permeability, and good deformation resistance. The oil-stone ratios of AC-13 and SAC-13 are 5.0% and 5.0%, and the target air voids are 6.7% and 6.5%, respectively.

### 2.2. MTS Compaction Process

In order to get the complex parameters of the asphalt mixture compaction process and the change law of the temperature field, and to ensure the uniform distribution of aggregate. The test was carried out using a 48 Hz vibration platform, a Pt100 + paperless recorder, and a MTS810 material test system. The test plan mainly simulates pavement compaction. The asphalt mixture mixing temperature is 160 °C and the indoor ambient temperature is controlled to be about 25 °C. The mixed asphalt mixture was placed in a test piece cylinder having a diameter of 150 mm and a temperature sensor (Pt100) was buried in the mixture during the charging process. The mold containing the asphalt mixture was placed on a vibrating table for 10 s, the vibration frequency was 48 Hz, and then the mold was placed on the MTS for loading. Two parallel tests were performed on the same type of asphalt mixture.

**Figure 3:**
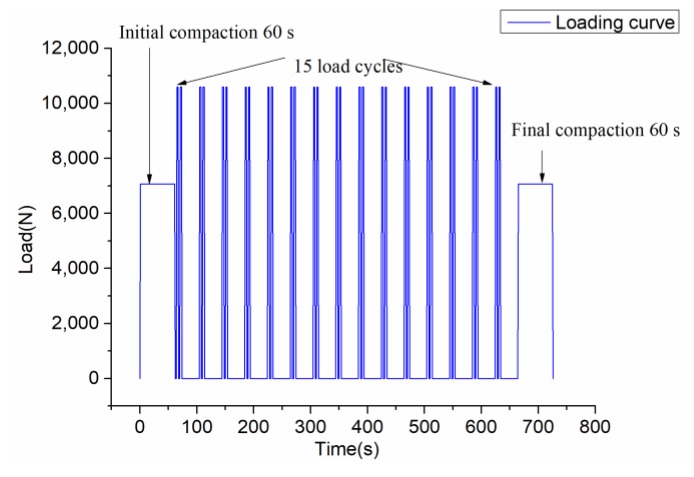
Loading curve and repeating cycles.

**Figure 4:**
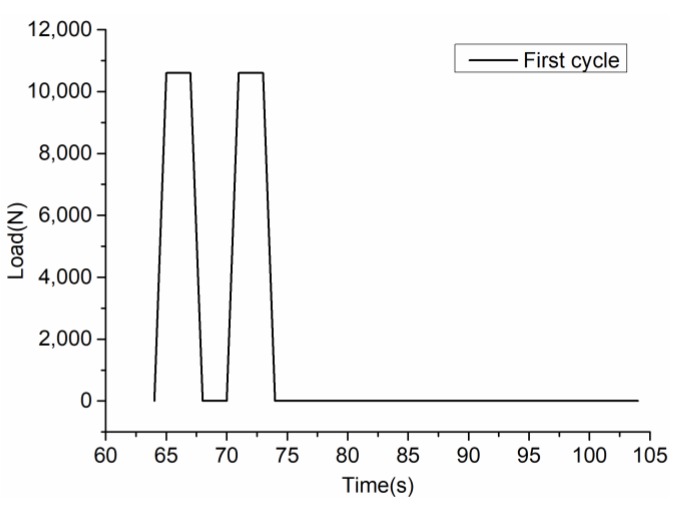
Loading curve of the first cycle.

To consider the rolling speed and the interval of the roller in a real construction project, Figure 3 shows the loading process. First compact for 60 s (initial compaction), then 15 cycles of loading (repeat compaction), and finally compact for 60 s (final compaction). The loading mode of cyclic load is: 1 s loading–2 s dead load–1 s unloading–2 s no load–1 s loading–2 s dead load–1 s unloading–30 s no load. Afterwards, repeat this cycle for 14 times. Figure 4 shows the first load cycle. Compaction load design considers SGC compaction load (generally 0.6 MPa) and relevant Chinese standards [23]. The initial and final compaction loads are 0.4 MPa, the repeated compaction loads are 0.6 MPa, and the total compaction time is 726 s.

The Pt100+ paperless recorder collects the temperature changes during the compaction process at a 1 s interval. The MTS computer is used to automatically record the pressure, time, and deformation during the test at a 0.005 s interval.

## 3. Viscoelastic-Plastic Rheological Model Theory

### 3.1. Nonlinear Viscoelastic-Plastic Nishihara Model and Parameter Solving

**Figure 5:**
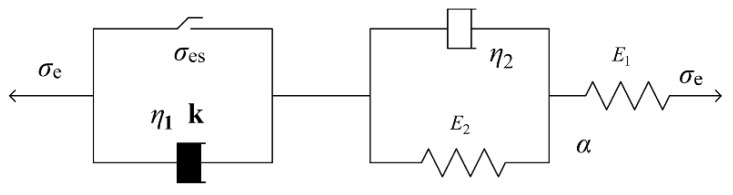
Nonlinear Nishihara model.

The Nishihara model is widely used to study the rheological properties of rocks [25,26,27,28,29,30]. However, it does not consider the effects of nonlinearity and temperature stress. A nonlinear Nishihara model is established to analyze the rheological characteristics of materials based on the theory of viscoelastic-plastic Nishihara model. The nonlinear characteristic uses the power function type $\varepsilon \left(t\right)=At^{k}$ (*A* is the test constant, *k* is the rheological index. Its value depends on material properties, stress levels, and temperature conditions), as shown in Figure 5.

When considering the influence of temperature stress and temperature strain on the stress and strain of the material, the constitutive equation of the nonlinear Nishihara model can be written as:

(1) $$\varepsilon \left(t\right)=\{\begin{array}{c} \sigma_{e}/E_{1}+\sigma_{e}/E_{2}\left[1-\exp(-E_{2}t/\eta_{2}\right)]+\alpha \cdot \Delta T\;(\sigma_{e}<\sigma_{e}{}_{S})\\ \sigma_{e}/E_{1}+\sigma_{e}/E_{2}\left[1-\exp(-E_{2}t/\eta_{2}\right)]+\left(\sigma_{e}-\sigma_{e}{}_{S}\right)t^{k}/\eta_{1}+\alpha \cdot \Delta T\;\left(\sigma_{e}\geq \sigma_{e}{}_{S}\right) \end{array}$$

where: *E_1_* is the elastic modulus of the Hooker body; *E_2_* is the elastic modulus of the Kelvin body; *η_1_*, *η_2_* are the viscosity coefficients of the Kelvin body and the Bingham body stick component; *σ_e_* is the applied stress; *σ_es_* is the material yield stress; *t* is time; *k* is the rheological index; Δ*T* is the calculation of the temperature difference; and, α is the material shrinkage coefficient.

The above formula fully considers the influence of factors, such as nonlinearity, time, and temperature stress of the material. Therefore, the model can more comprehensively reflect the nonlinear viscoelastic-plastic properties of the material.

The material is subjected to compressive stress and it takes a negative value, and the compaction of the hot mix asphalt mix is generally completed above 100 °C, so the asphalt mixture is in a plastic flow dynamic, and the nonlinear viscous property is approximately linear viscous behavior, that is to take k≈1; at the same time, the temperature shrinkage stress $\sigma_{T}=E\alpha \cdot \Delta T\approx 0$ MPa (where $E=u\times 10^{3}\;$MPa, ΔT≈0.1 °C, Δ*T* is the within 2s loading temperature drop range). In addition, studies have shown that the higher temperature asphalt mixture has good stress relaxation performance, and the temperature stress that is generated by cooling can quickly relax to zero [31]. Therefore, the asphalt mixture pressure has no temperature stress in real time. 

The constitutive equation of the nonlinear rheological model of the hot mix asphalt compaction process can be simplified as:

(2) $$\varepsilon \left(t\right)=-\begin{array}{cc} \left\{\sigma_{e}/E_{1}+\sigma_{e}/E_{2}\left[1-\exp(-E_{2}t/\eta_{2}\right)]+\left(\sigma_{e}-\sigma_{e}{}_{S}\right)t/\eta_{1}\right\} & \left(\sigma_{e}\geq \sigma_{e}{}_{S}\right) \end{array}$$

where: *E_1_* is the elastic modulus of the Hooker body; *E_2_* is the elastic modulus of the Kelvin body; *η_1_*, *η_2_* are the viscosity coefficients of the Kelvin body and the Bingham body stick component; *σ_e_* is the applied stress; *σ_es_* is the material yield stress; and, *t* is time.

Equation (2) can ignore the influence of temperature effects during the calculation of stress and strain. It not only simplifies the model theory, but it also makes the determination of model parameters simpler and easier. The meaning of all parameters is shown in Table A1.

### 3.2. Model Parameter Solving

In order to simulate the road compaction process, each load cycle includes two loading and unloading. It is assumed that the temperature is the same in one cycle, but different for different cycle. In Equation (2), there are six model parameters of *σ_e_, σ_es_, E_1_, E_2_, η_1_,* and *η_2_*. Each parameter is a related parameter that varies with the number of load cycles and temperature.

**Figure 6:**
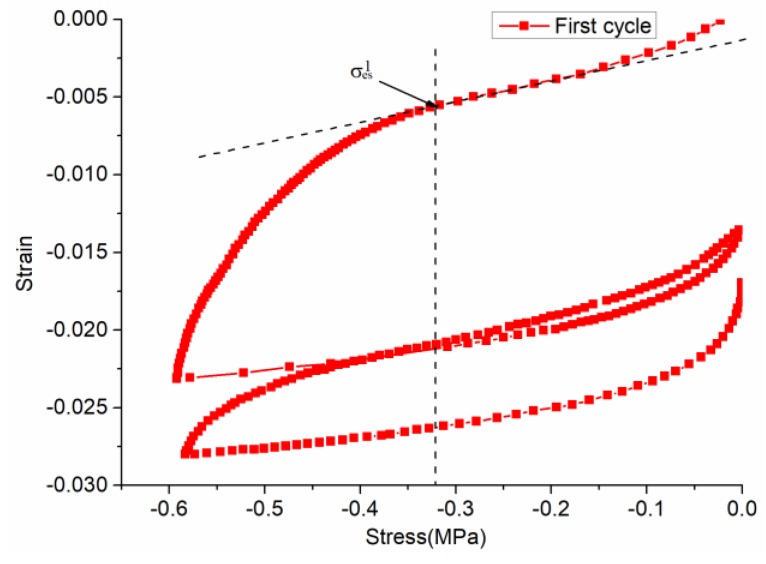
The first cyclic stress–strain curve of SAC-13.

In the analysis of viscoelastic-plastic parameters, the first loading process curve, the second unloading process curve, and the whole viscoplastic deformation in 15 loading cycles are analyzed. Combining the rheological model constitutive equation and the nonlinear regression equation, the solution ideas of each model parameter are presented, as follows:

(1) $\sigma_{e}\approx \sigma_{0}=0.6\;\mathrm{Mpa}$.
(2) Under the action of 15 loading cycles, the value of *σ_es_* in each cycle is during two loading and unloading processes. The tangent stress value at the straight line segment of the stress-strain curve at the first loading and the tangent point at the curve is taken as the *σ_es_* of the cyclic process, as shown in Figure 6.
(3) Separating viscoplastic strain, elastic strain, and viscoelastic strain solves the parameters. Firstly, *E_1_* is solved by the “recovery elastic strain” after the second unloading in each cycle: $E_{1}=\sigma_{e}/\varepsilon^{e}$. Secondly, the *η_1_* value is solved by the “median viscoplastic strain” in each cycle:$\;\eta_{1}=2\left(\sigma_{e}-\sigma_{e}{}_{S}\right)t/\varepsilon^{vp}\left(t\right)$. Thirdly, in the Kelvin body, *E_2_* and *η_2_* use the BoxLucas1 model equation $y=a\left(1-e^{-bx}\right)$ to perform nonlinear regression on the “recover viscoelastic strain time curve” after the second unloading in each cycle, and the parameters of the model are then obtained by calculating.

The stress–strain–time response curves of the SAC-13 matrix asphalt mixture in the first cycle are taken as an example to solve the model parameters. Table 5 shows the viscoelastic plastic strain results of the SAC-13 asphalt mixture.

**Figure 7:**
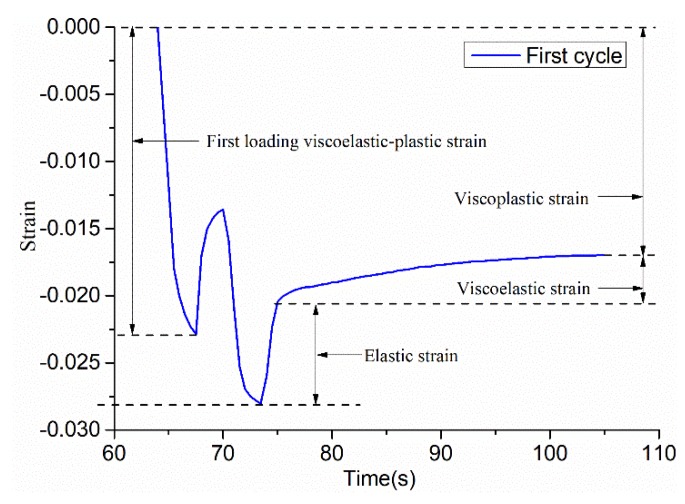
The first cycle strain–time curve of SAC-13.

**Figure 8:**
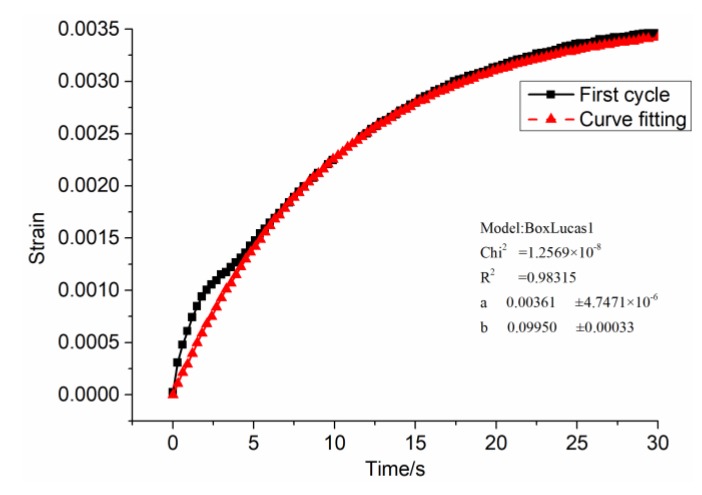
Nonlinear regression of the first cycle viscoelastic strain time curve of SAC-13.

The first step is to solve the yield stress *σ_es_*. Through the first cyclic stress–strain curve of Figure 6, the value of the tangent point at the straight line segment of the stress–strain curve at the first loading and the tangent point of the curve is determined, and $\sigma_{es}^{1}$ is obtained. In the same way, $\sigma_{es}^{2}$ ⋯ $\sigma_{es}^{15}$ can be obtained. The second step is to solve *E_1_, E_2_, η_1_,* and *η_2_*. The elastic strain, viscoplastic strain, and viscoelastic strain have been marked in Figure 7, and the parameters of the table are solved, as follows:

(a) $E_{1}=\sigma_{e}/\varepsilon^{e}$, then $E_{1}^{1}=0.6/\varepsilon^{e1}=0.6/0.00753=79.681$, in the same way, $E_{1}^{2}$⋯ $E_{1}^{15}$ can be obtained.
(b) $\eta_{1}=2\left(\sigma_{e}-\sigma_{e}{}_{S}\right)t/\varepsilon^{vp}\left(t\right)$, then $\eta_{1}^{1}=\left(\sigma_{e}-\sigma_{\mathrm{eS}}^{1}\right)\times 2/\left(\varepsilon^{vp1}/2\right)$, that is $\eta_{1}^{1}=\left(0.6-0.340\right)\times 2/\left(0.01747/2\right)=64.34$, and in the same way, $\eta_{1}^{2}$*⋯*$\eta_{1}^{15}$ can be obtained.
(c) The BoxLucas 1 model equation $y=a\left(1-e^{-bx}\right)$ is used for non-linear regression, as shown in Figure 8. Where $a=\sigma_{e}/E_{2}$, $b=E_{2}/\eta_{2}$. After calculation, $E_{2}^{1}=0.6/0.00231=166.205$, $\eta_{2}^{1}=166.205/0.09950=1670.4$. In the same way, $E_{2}^{2}$⋯ $E_{2}^{15}$ and $\eta_{2}^{2}$⋯ $\eta_{2}^{15}$ can be obtained.

## 4. Nishihara Model Verification and Parameter Analysis

### 4.1. Model verification

**Figure 9:**
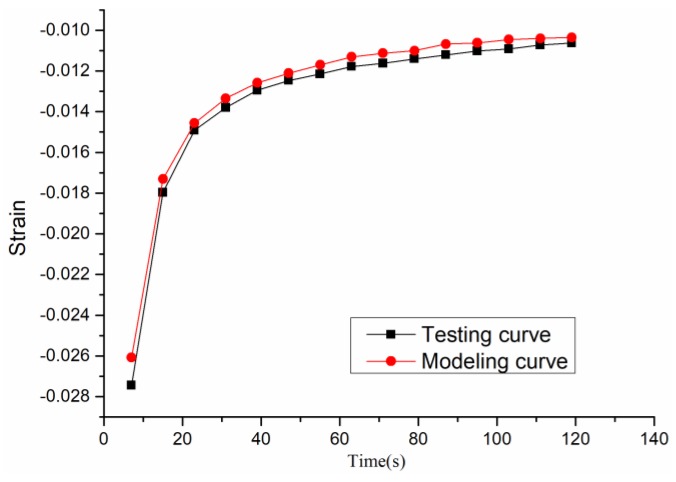
Testing curve and modeling curve in a Material Testing System (MTS) compaction test.

The six model parameters *σ_e_, σ_es_, E_1_, E_2_, η_1_,* and *η_2_* are substituted into Equation (2) to obtain the strain curves when compared with the testing strain–time curves, as shown in Figure 9.

It can be seen that the flow before the third load cycle is obvious, because the early asphalt mixture is not compact enough. After the third load cycle, the deformation begins to decrease as the number of load cycles increases, because the asphalt mixture tends to be dense. It can be found the calculated value is slightly smaller than the experimental value, but the basic change law of the compression test can be simulated, and the difference between the two should be caused by the difference between the test pieces. This proves that the viscoelastic theory is used to simulate the rheological properties of hot mix asphalt in the compaction process with certain rationality and effectiveness.

### 4.2. Parameter Analysis

The model parameter values were calculated by nonlinear regression analysis of the response curve of the asphalt mixture. In this paper, Table 6 shows only the parameter values calculated for the fifteenth load cycle. 

**Figure 10:**
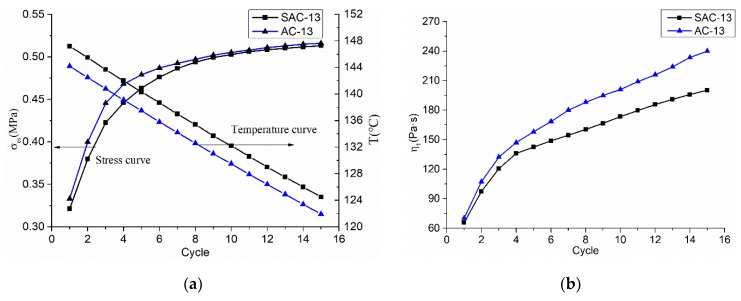
Model parameters and temperature curves with load cycle times; (**a**) *σ_es_* and T curves with load cycle times; and, (**b**) *η_1_* curves with the load cycle times.

The viscoplastic viscous coefficient *η_1_* characterizes the viscous effect of the viscoplastic process of the mixture, which reflects the ability of the asphalt mixture to resist deformation during the viscoplastic phase. Figure 10 indicates that, as the number of loading cycle increases, the temperature of the mixture decreases, and the yield stress and viscosity coefficient increase. The viscosity coefficient mainly increases because the viscosity of the asphalt increased when the temperature decreases. The rate change of yield stress and viscosity coefficient of the real-time mixture is faster and the mixture is easily compacted. After the third load cycle, the rate of change of yield stress and viscosity coefficient of AC-13 began to decrease. After the fourth load cycle, the rate of change of yield stress and viscosity coefficient of SAC-13 also began to decrease, and the mixture began to become more difficult to compact. 

**Figure 11:**
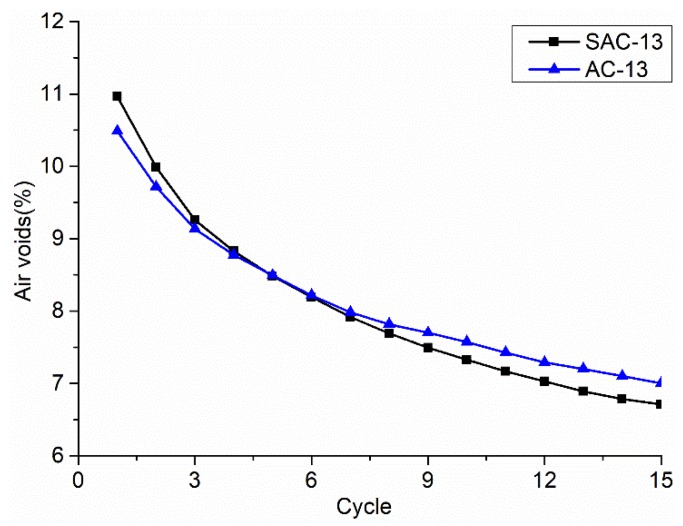
Air voids curves changing with the load cycle times.

In Figure 11, as the number of load cycles increases, the air voids decreases. SAC-13 contains more coarse aggregates than AC-13 and the theoretical air voids should be relatively large. However, it can be seen that, as the number of load cycles increases, the air voids of SAC-13 begins to be less than AC-13. This shows that, the more coarse aggregate, the air voids is not necessarily greater, and the mixture is not necessarily harder to compact. The tendency of air void curves implies that AC-13 was becoming more difficult to be compacted. 

**Figure 12:**
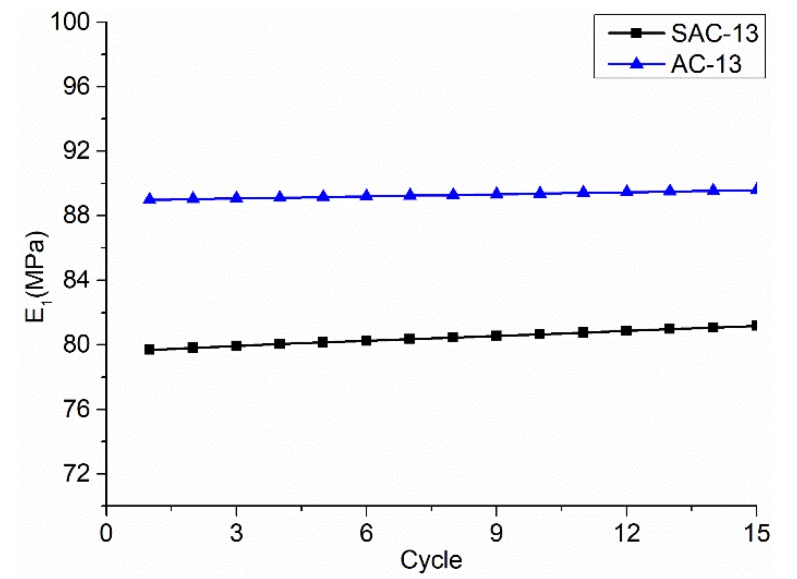
Elastic parameters curves changing with the load cycle times.

*E_1_* is the elastic modulus, which is known as an index to measure the instantaneous difficulty of elastic deformation of materials. The larger the value, the greater the ability to resist deformation. Figure 12 presents that *E_1_* for SAC-13 stable as the number of load cycles changes, because the asphalt cement has strong fluidity at high temperature, and the elastic modulus of the mixture is mainly expressed as the elastic modulus of the aggregate skeleton in this state. The difference in the elastic modulus of the two grades is mainly due to the large amount of coarse aggregate and the high void ratio.

**Figure 13:**
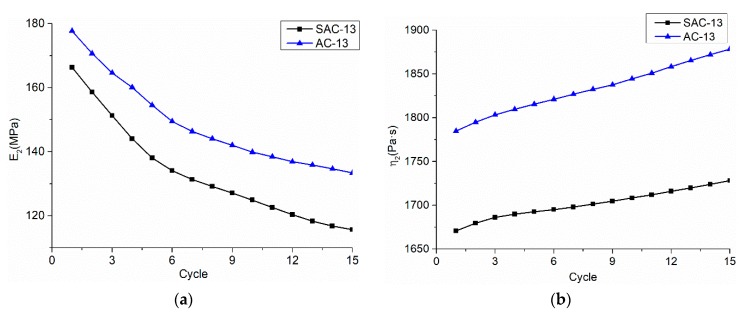
Viscoelastic parameters change with the load cycle times; (**a**) *E_2_* change with the load cycle times; and, (**b**) *η_2_* change with the load cycle times.

*E_2_* is the viscoelastic deformation modulus, which characterizes the viscoelastic recovery effect of the mixture. It can be seen from Figure 13 that, as the number of load cycles increases, *E_2_* decreases, and its ability to resist deformation decreases. The viscoelastic viscosity coefficient *η_2_* characterizes the viscous effect of the viscoelastic process of the mixture, which reflects the ability of the asphalt mixture to resist the deformation during the viscoelastic phase. Figure 13 presents that *η_2_* becomes larger as the number of load cycles increases, and the ability of the mixture to resist deformation becomes larger. The change in the two parameters is because, as the number of load cycles increase, the temperature decreases and the viscosity of the asphalt decreases. The difference in viscosity coefficients between the two grades is due to temperature differences and gradation differences, it can be summarized that the increasing rate of *η_2_* for AC-13 proves the difficulty in compaction.

## 5. Conclusion

Based on the common Nishihara model, this paper establishes a constitutive equation for the nonlinear Nishihara model by considering the influence of nonlinearity and temperature stress of asphalt mixture. The MTS compaction test obtained the stress–strain curve and the time–strain curve, and the viscoelastic-plastic parameters were solved on this basis. Finally, the model parameters were substituted into the constitutive equation to verify, and the viscoelastic-plastic parameters of the two graded asphalt mixtures are analyzed.

1. The nonlinear Nishihara model has the advantages of being intuitive, simple in theory, and possessing easy to solve parameters. A loading and unloading MTS compaction test is effectively utilized to calculate the parameters. The calculation curve is compared with the test curve, and it is verified that the rheological properties of the hot mix asphalt in the compaction process can be indirectly characterized by the nonlinear Nishihara model.
2. The results prove that the nonlinear Nishihara model is useful in distinguishing the flow behavior undergoing a load and unloading compaction. The compaction of asphalt mixture mainly produces plastic deformation, which is mainly manifested by changes in viscos and plastic parameters (*σ_es_*, *η_1_*, and *η_2_*), rather than the elastic parameter (*E*_1_). The differences in sensitive parameters (*σ_es_*, *η_1_*, and *η_2_*) reflect that the gradation and temperature have certain influence on the compaction characteristics of the asphalt mixture.
3. Only two asphalt mixtures and one stress were included in this paper. The next step will be aiming at studies regarding the compaction properties subjected to various grades, different modified asphalts, and stress at different temperatures via the Nishihara model.

## Figures and Tables

**Table 1:** Physical Properties of 70# matrix asphalt.

Test Items		Value	Required
Penetration (25 °C, 100g, 5s)(0.1mm)		65	60–80
Ductility	(5 cm/min, 10 °C) cm	86	≥20
	(5 cm/min, 15 °C) cm	>100	≥100
Softening Point TR&B (°C)		50	≥46
Penetration index		−0.476	−1.5–+1.0
60 °C Dynamic viscosity (Pa·S)		224	≥180
Wax content (%)		1.2	≤2.2
Solubility (%)		99.8	≥99.5
Flash point (°C)		330	≥260
Density 15 °C (g/cm^3^)		1.012	Measured
After RTFOT	Quality change (%)	−0.026	≤±0.8
	Residual penetration ratio (%)	63	≥61
	Residual ductility (10 °C) (cm)	7	≥6
	Residual ductility (15 °C) (cm)	32	≥15

**Table 2:** Physical Properties of Coarse Aggregates.

Test Items	Value			Required
	13.2–9.5	9.5–4.75	4.75–2.36	
Apparent relative density	2.859	2.822	2.769	≥2.6
Water absorption (%)	0.52	0.57	0.73	≤2
Crushing value (%)	21	--	--	≤26
Los Angeles abrasion (%)	19.2	--	--	≤28

**Table 3:** Physical Properties of Fine Aggregates.

Test Items	Value	Required
Apparent relative density	2.717	≥2.5
Mud content (percent of < 0.075 mm) (%)	0.9	≤3
Sand equivalent (%)	83.7	≥60
Angularity	40.3	≥30

**Table 4:** Physical Properties of Mineral Filler.

Test Items		Value	Required
Apparent relative density (g/cm^3^)		2.638	≥2.5
Water absorption (%)		0.3	<1
Grain sizes (%)	<0.6 mm	100	100
	<0.15 mm	99.9	90–100
	<0.075 mm	90.2	75–100
Hydrophilic coefficient		0.62	<1

**Table 5:** Viscoelastic-plastic strain of AC-13 and SAC-13 asphalt mixture.

Noumber	SAC-13		
	Viscoplastic Strain (*ε^vp^*)	Elastic Strain (*ε^e^*)	Viscoelastic Strain (*ε^ve^*)
First	−0.017470	0.007530	0.0034020
Second	−0.008624	0.007504	0.0036927
Third	−0.005866	0.007503	0.0038537
Fourth	−0.004640	0.007496	0.0039342
Fifth	−0.003839	0.007494	0.0041183
Sixth	−0.003410	0.007491	0.0042349
Seventh	−0.003276	0.007491	0.0040201
Eighth	−0.002605	0.007439	0.0042196
Ninth	−0.002484	0.007435	0.0041720
Tenth	−0.002362	0.007421	0.0042173
Eleventh	−0.002047	0.007420	0.0043321
Twelfth	−0.001987	0.007418	0.0043233
Thirteenth	−0.001820	0.007417	0.0044842
Fourteenth	−0.001779	0.007401	0.0045524
Fifteenth	−0.001740	0.007391	0.0043724

**Table 6:** Parameters of the 15th load cycle.

Mix type	*T* (°C)	Air Voids (%)	Value					
			*σ_e_*	*σ_es_*	*E_1_*	*E_2_*	*η_1_*	*η_2_*
			(MPa)	(MPa)	(MPa)	(MPa)	(Pa·s)	(Pa·s)
SAC	124.5	6.7	0.6	0.513	81.18	115.83	200.041	1728.016
AC	121.9	7.0	0.6	0.516	89.634	133.311	241.371	1877.990

## Appendix A

**Table A1:** Parameter comment table.

***σ_e_***	Applied stress	***ε^e^***	Elastic strain
***σ_es_***	Yield stress	***ε^ve^***	Viscoelastic strain
***E_1_***	The elastic modulus of the Hooker body	***k***	The rheological index
***E_2_***	The elastic modulus of the Kelvin body	***t***	Time
***η_1_***	The viscosity coefficients of the Kelvin body stick component	***α***	The material shrinkage coefficient
***η_2_***	The viscosity coefficients of the Bingham body stick component	**Δ*T***	The value of the temperature difference
***ε^vp^***	Viscoplastic strain		
